# Seeing Red: Anger Increases How Much Republican Identification Predicts Partisan Attitudes and Perceived Polarization

**DOI:** 10.1371/journal.pone.0139193

**Published:** 2015-09-25

**Authors:** Michaela Huber, Leaf Van Boven, Bernadette Park, William T. Pizzi

**Affiliations:** 1 Department of Psychology, Technische Universitaet Dresden, Dresden, Germany; 2 Department of Psychology and Neuroscience, University of Colorado Boulder, Boulder, Colorado, United States of America; 3 Colorado Law School, University of Colorado Boulder, Boulder, Colorado, United States of America; Virginia Commonwealth University, UNITED STATES

## Abstract

We examined the effects of incidental anger on perceived and actual polarization between Democrats and Republicans in the context of two national tragedies, Hurricane Katrina (Study 1) and the mass shooting that targeted Representative Gabrielle Giffords in Arizona (Study 2). We hypothesized that because of its relevance to intergroup conflict, incidental anger exacerbates the political polarization effects of issue partisanship (the correlation between partisan identification and partisan attitudes), and, separately, the correlation between conservative partisan identification and perceived polarization between Democrats and Republicans. We further hypothesized that these effects would be strongest for Republican identification because Republican leaders were targets of public criticism in both tragedies and because conservative (Republican) ideology tends to be more sensitive to threat. In the studies, participants first completed an emotion induction procedure by recalling autobiographical events that made them angry (Studies 1 & 2), sad (Studies 1 & 2), or that involved recalling emotionally neutral events (Study 2). Participants later reported their attitudes regarding the two tragedies, their perceptions of the typical Democrat’s and Republican’s attitudes on those issues, and their identification with the Democratic and Republican parties. Compared with incidental sadness (Studies 1 and 2) and a neutral condition (Study 2), incidental anger exacerbated the associations between Republican identification and partisan attitudes, and, separately between Republican identification and perceived polarization between the attitudes of Democrats and Republicans. We discuss implications for anger’s influence on political attitude formation and perceptions of group differences in political attitudes.

## Introduction

The Pew Research Center, a nonpartisan research center based in the U.S., recently reported that the divide between Democrats and Republicans in the United States is at its highest level in more than 25 years [1]. Americans are not only increasingly polarized, they are also increasingly *perceived* as politically polarized [2,3]. Both the actual and perceived divide along political party lines exacerbate political gridlock, pose barriers to civil dialogue, and contribute to a political environment filled with vitriol and mutual distrust. Political polarization is thus an emotionally charged and salient component of contemporary social life. Because identification with political groups is a central component of social identity [4], and because political groups (e.g. political parties) constitute an important part of social life, understanding the nature of partisan attitudes and perceptions of partisan attitudes is an important facet of everyday social cognition.

We suspected that incidental anger might moderate partisan attitudes and perceptions of partisan attitudes. We examined the effects of incidental anger because emotions often pervade political discourse, shaping and reflecting political divisions and partisan attitudes, and because anger, in particular, is associated with intergroup relations and conflict. In general, incidental emotions profoundly shape social cognitions and behaviors, including intergroup attitudes [5], interpersonal trust [6], confidence [7], economic valuation [8], and information processing more generally [9]. Anger is often associated with intergroup conflict, and is associated with people’s intentions to aggress against the out-group (e.g.,[10]). We hypothesized that incidental anger would increase the correlation between partisan identification and partisan attitudes [11], and would increase the correlation between partisan identification and perceived polarization. The possibility that incidental anger influences partisan attitudes and perceived polarization would provide novel evidence regarding the constructed nature of partisan attitudes, and regarding how emotions, even when incidental in nature, influence political-social-cognition.

We tested these predictions in regards to two national tragedies: Hurricane Katrina and its aftermath in 2005, and the 2011 mass shooting involving Representative Gabrielle Giffords in Tucson, Arizona. We chose these national tragedies because both were salient social-political events that were sources of conflict between Democrats and Republicans. Both Hurricane Katrina [12] and the Giffords shooting were significant flashpoints of political debate.

We derived our key hypotheses from two postulations, elaborated below, that are well established by previous research. First, we suggest identity-based concerns about maintaining the integrity of the political group people identify with will increase endorsement of partisan attitudes and perceptions of distinctiveness—which results in perceived polarization in partisan contexts—between political groups. That is, the more people identify with a political group (i.e. political party), particularly when that political group is threatened by the specific event, the more people will adhere to partisan positions, and the more people will perceive political groups as adhering to those positions. Second, due to the association between anger and intergroup conflict, incidental anger should exacerbate these identity-based concerns, resulting in even greater polarization of partisan attitudes and even greater perceived polarization.

### Identification, Issue Partisanship, and Perceived Polarization

Social identity theory implies that to the degree people strongly identify with a group, they tend to perceive the social world in a way that protects their group’s distinctiveness and integrity. We examined two perceptions that reflect and foster group distinctiveness and integrity. First, to the degree that people identify with a political party, they adopt and express attitudes associated with that party [13–16]. Political scientists refer to this pattern as issue partisanship, reflecting that people hold attitudes that correspond with their partisan identification [3,11]. Following Hurricane Katrina, not surprisingly, Republicans expressed more favorable evaluations of the Republican Administration’s response than did Democrats [12]. And following the 2011 mass shooting in Arizona, Republicans were less inclined than Democrats to agree with charges that a prominent Republican politician’s “hateful” political rhetoric cultivated a violent climate that fostered the shooting [17,18]. Social identity concerns thus contribute to issue partisanship [19]. The second type of perception that corresponds with group distinctiveness is the perception of polarization between the political groups, specifically between Democrats and Republicans [3]. People with stronger political party identification perceive greater differences between partisan groups [3,20–23]. Such polarization perception affirms the distinctiveness of people’s own group from the opposing group [24–27].

In our studies, we measured partisan identification by asking respondents how much they identified with the Democratic Party and with the Republican Party on two separate scales. Emerging evidence indicates that, just as with other forms of social identification, political identification is a continuous multi-dimensional construct. People can simultaneously identify (or dis-identify) somewhat with both the Democratic and Republican Parties [28,29]. Measuring identification with each political party separately allowed us to examine the effects of identification simultaneously with both parties without forcing a tradeoff between identification with the two. In contrast to this approach, researchers often measure political identification on a single scale with endpoints labeled Democrat and Republican. Such bipolar measures confound identification with one party and disidentification with the other party; such measures implicitly assume a perfect negative correlation between the two identities. Bipolar measures also do not allow intensity ambivalence, where people identify (or dis-identify) equally with both parties.

Given that both issue partisanship (the correlation between partisan identification and partisan attitudes) and partisan perceived polarization (the correlation between partisan identification and perceived polarization) serve in part to protect the group they identify with, we reasoned that both tendencies would be pronounced for identification with a threatened group. In our studies, we predicted that both tendencies would be more pronounced for identification with the Republican Party and not for identification with the Democratic Party because the Republican Party was threatened and not the Democratic Party. There are two reasons for this prediction: First, the events we examine, Hurricane Katrina and the Giffords shooting, involved situational contexts where Republican Party leaders (President Bush and Governor Sarah Palin) were under attack. [17]. Second, ideological conservatives tend to identify with the Republican Party, and conservative ideologies are associated with heightened defensiveness and threat sensitivity [30,31]. We therefore examined issue partisanship and partisan perceived polarization as a function of Republican identification, controlling for Democratic identification. We will return to this issue in the General Discussion, but one untested assumption of the current studies and a question for future research is to examine issue partisanship and partisan perceived polarization in contexts where leaders of the Democratic Party were criticized. In those contexts, issue partisanship and partisan perceived polarization should be examined as a function of Democratic identification and Republican identification.

### Incidental Anger

Emotions profoundly influence intergroup relations (e.g., [10,32]). Anger, in particular, is associated with intergroup conflict [10,32,33], including political conflict (see also, [34]). Anger toward partisan opponents fosters aggressive actions and endorsement of aggressive policies toward opposing political groups [35–37]. Anger is associated with intergroup threats even when those threats do not directly involve the self [38]. Anger has also been viewed as an emotion associated with a motivational tendency to correct the situation that aroused the anger [39]. In an intergroup context, this situation could be an injustice done by the out-group that needs to be corrected [40]. Anger is therefore associated with intergroup conflict, threat, and perceived wrongdoing by opposing groups. Given the association between anger and intergroup conflict, social cognitive tendencies that are triggered by intergroup conflict—such as issue partisanship and perceived polarization—should be increased when people are angry.

Most research on anger and intergroup conflict has examined integral emotions, those that are aroused by explicit appraisals of conflicted groups. However, it is difficult to directly connect such effects to emotional states because integral emotions directly and explicitly influence people’s appraisal of the opposing group. For example, making Republicans angry at Democrats for unfairly blaming President Bush for ineptly overseeing the delivery of aid following a natural disaster necessarily entails a host of different mental representations compared to making Republicans sad about the loss of life, culture, and infrastructure in New Orleans, LA, following Hurricane Katrina.

Researchers have addressed these interpretational ambiguities regarding integral emotions by examining the effects of incidental emotions, those aroused by contexts that are not explicitly related to the topic at hand. Most relevant for our hypotheses, researchers have shown that incidental anger increases implicit intergroup bias [5,41]. People who described personal events that had made them angry exhibited larger implicit intergroup biases toward minimally defined groups of “over-estimators” and “under-estimators” compared with people who described personal events that made them sad or neutral [5]. Based on findings such as these, we hypothesized that incidental anger would increase issue partisanship and perceived polarization to the degree that people identified as Republican.

In our studies, we compared the effects of incidental anger with the effects of incidental sadness (cf., [5,41–43]). Sadness is an ideal comparison to anger because sadness is a negative emotion—one that people often experience in response to the kinds of national tragedies we examine—that is less relevant to intergroup conflict [5]. Also, both anger and sadness are negative emotions that are oriented to past events, whereas other negative emotions such as fear are oriented to future events [44].

### Aim and Hypotheses

In sum, we had two hypotheses. Hypothesis 1 was that incidental anger would increase the correlation between people’s identification with the Republican Party and their partisan attitudes (issue partisanship). Hypothesis 2 was that incidental anger would increase the correlation between Republican identification and perceived polarization between the attitudes of Democrats and Republicans (partisan perceived polarization).

We tested these hypotheses in the context of two real-world events, with diverse samples of respondents. In Study 1, participants evaluated the Bush Administration’s handling of Hurricane Katrina. In Study 2, participants evaluated Governor Sarah Palin’s role in fostering violence prior to the Giffords shooting in Arizona. We also asked participants in both studies to estimate the attitudes of typical Democrats and typical Republicans. In both studies, we expected and found that incidental anger increased the correlation between the degree to which people identified as Republican and their endorsement of pro-Republican attitudes (issue partisanship), and the correlation between Republican identification and perceived polarization between Democrats and Republicans (partisan perceived polarization).

## Study 1: Hurricane Katrina

We conducted the study in the aftermath of Hurricane Katrina, a storm that caused severe destruction on the Gulf coast of the U.S. In the aftermath of the hurricane, the federal government under Republican leadership faced much public criticism and outrage for their slow and inadequate response. We reasoned that this outrage directed at a Republican, namely President George W. Bush, threatened people to the degree that they identify with the Republican Party.

We experimentally manipulated participants’ incidental anger or sadness in a community sample before asking them to evaluate the Bush Administration’s handling of Hurricane Katrina, to estimate the evaluations of typical Democrats and Republicans, and to report how much they identified as a Democrat and Republican. We predicted that, compared with incidental sadness, anger would increase the associations of strength of identification with the Republican Party with (1) positive evaluations of the Bush Administration and with (2) perceived polarization between Democrats and Republicans. We expected these two effects to be independent of each other.

### Method

Participants (*N* = 115, 57% female) approached in public places in Denver and Boulder, CO, between 6 March and 28 April 2006 participated in exchange for $10, completing the study on laptop computers while wearing noise-canceling headphones. The Institutional Review Board at the University of Colorado Boulder approved the study. Participants provided written informed consent. Participants varied in age (*M* = 41.94 years, *SD* = 12.94, IQR = 31–53 years [IQR refers to the interquartile range, covering the range from the 25^th^ to 75^th^ percentile.]) and ethnicity (White = 56%, Hispanic = 31%, Native American = 4%, Asian American = 3%, African American = 2%, other = 4%).

To manipulate incidental emotion, we randomly assigned participants “to describe up to five events or situations that made you feel very” angry (*n* = 52) or sad (*n* = 63) (cf., [5,42]). As part of an ostensibly unrelated task, participants then read that we were “interested in your reactions to national events… in your judgments about various policies, social issues, and government entities related to Hurricane Katrina.” To measure evaluation of the Bush Administration, participants indicated agreement (1 = *strongly disagree*; 7 = *strongly agree*) with two statements: “Overall, President Bush did a good job in handling relief efforts in response to the hurricane,” and “Overall, the federal government under the Bush administration did a good job in handling relief efforts in response to the hurricane,” which we averaged (*r* = .69) into a measure of Bush Administration evaluation.

We then asked participants to estimate, separately and in counterbalanced order, how much the typical Republican and the typical Democrat would agree with each of the two statements. We averaged these ratings into indices of perceived evaluations of the Bush Administration by the typical Republican (*r* = .64) and by the typical Democrat (*r* = .78). We subtracted participants’ estimates of the typical Democrat’s response (*M* = 1.87, *SD* = 1.31) from their estimates of the typical Republican’s response (*M* = 5.06, *SD* = 1.46) such that higher numbers reflect greater perceived polarization.

**Fig 1:**
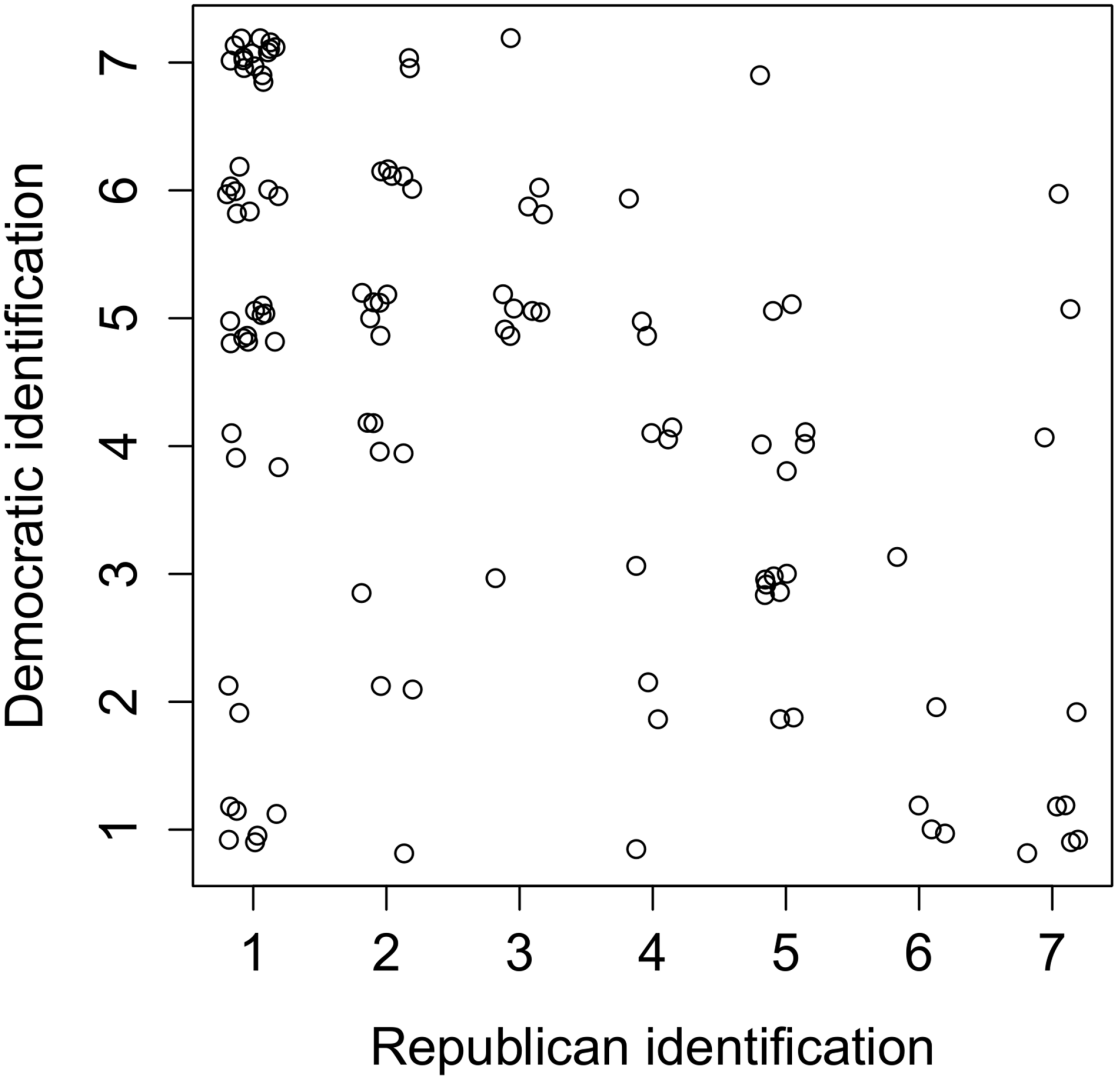
Study 1: Scatterplot displaying Republican and Democratic identification. The scatterplot displays variance on Republican identification (x-axis) and Democratic identification (y-axis) in the sample for Study 1 (*N* = 115).

To measure strength of political identification, participants reported, separately, how strongly they identified with the Republican and Democratic parties (1 = *not very strong*, 7 = *very strong*). Note that although these identifications were negatively correlated (*r* = –.47), the correlation was far from perfect, supporting the decision to measure and examine these as separate constructs (see Fig 1). Participants identified more with the Democratic (*M* = 4.37, *SD* = 1.98) than Republican Party (*M* = 2.84, *SD* = 1.99), *t*(114) = 4.79, *p* < .001, *d* = .45. Keep in mind that our predictions were about the strength of the association between Republican identification and outcome measures (evaluations of the Bush Administration and perceived polarization between Democrats and Republicans). To test the effect of emotion on the strength of these associations, it was important that our sample had respondents across the full spectrum of Republican identification (see Fig 1). Finally, participants were thanked and debriefed.

### Results and Discussion

The authors confirm that all data underlying the findings are fully available without restriction. We confirm that we reported all conditions and data exclusions. We collected additional measures that were not directly related to the hypotheses we tested here. All measures are included in the supplementary material. The sample size for this study was based on how much data we could afford to collect. Data may be accessed from Figshare under the DOI: http://dx.doi.org/10.6084/m9.figshare.1464978.

We regressed participants’ evaluation of the Bush Administration on Republican identification (mean centered), emotion condition (anger = +1, sadness = –1), the Republican identification × emotion condition interaction, Democratic identification, and perceived polarization (operationalized as described above, see Table 1). [Consistent with other research, we included perceived polarization in the model estimating participants’ own attitudes and we included participants’ own attitudes in the model estimating perceived polarization because the two constructs tend to be associated [3,23]. Here, the two constructs are negatively correlated, *r* = –.24. We excluded data from one participant from the analyses. In the linear regressions described, the outlier’s studentized deleted residual had a value of 4 and also displayed a large value on cook’s d. We applied the same exclusion criterion in Study 2.] Because our hypotheses focused on Republican identification, we controlled for Democratic identification to partial out effects of dis-identification with Democratic identification. Not surprisingly, there was a positive association between Republican identification and evaluation of the Bush Administration, *b* = .30, *t*(108) = 4.79, *p* < .001, partial η^2^ = .17, and a negative association between Democratic identification and evaluation of the Bush Administration, *b* = –.28, *t*(108) = –4.32, *p* < .001, partial η^2^ = .15.

**Table 1:** Study 1: Linear regression models.

*Dependent Variables and* Predictors	*b*	*SE(b)*	*t*	*p*	95% CI	partial η^2^
*Evaluation of Bush Administration*						
Emotion condition	–.13	.11	–1.14	.253	–.34, .03	.01
Republican identification (centered)	.30	.06	4.79	< .001	.17, .42	.17
Emotion × Republican identification	.12	.06	2.21	.029	.01, .23	.04
Democratic identification	–.28	.06	–4.32	< .001	–.40,–.15	.15
Perceived polarization	–.11	.05	–2.08	.040	–.22,–.01	.04
*Perceived Polarization*						
Emotion condition	–.07	.19	–0.38	.698	–.45, .31	.01
Republican identification (centered)	.12	.12	1.06	.294	–.11, .36	.01
Emotion × Republican identification	.20	.10	2.07	.040	.01, .39	.04
Democratic identification	.12	.12	0.97	.334	–.12, .35	.01
Evaluation of Bush Administration	–.34	.16	–2.08	.040	–.66,–.02	.04

*Linear regression models estimating evaluation of the Bush Administration’s response to Hurricane Katrina (top panel) and perceptions of polarization between Democratic and Republican evaluation of the Bush Administration response to Hurricane Katrina (bottom panel)*. Republican identification is mean centered. Emotion condition: anger = +1, sadness = –1.

**Fig 2:**
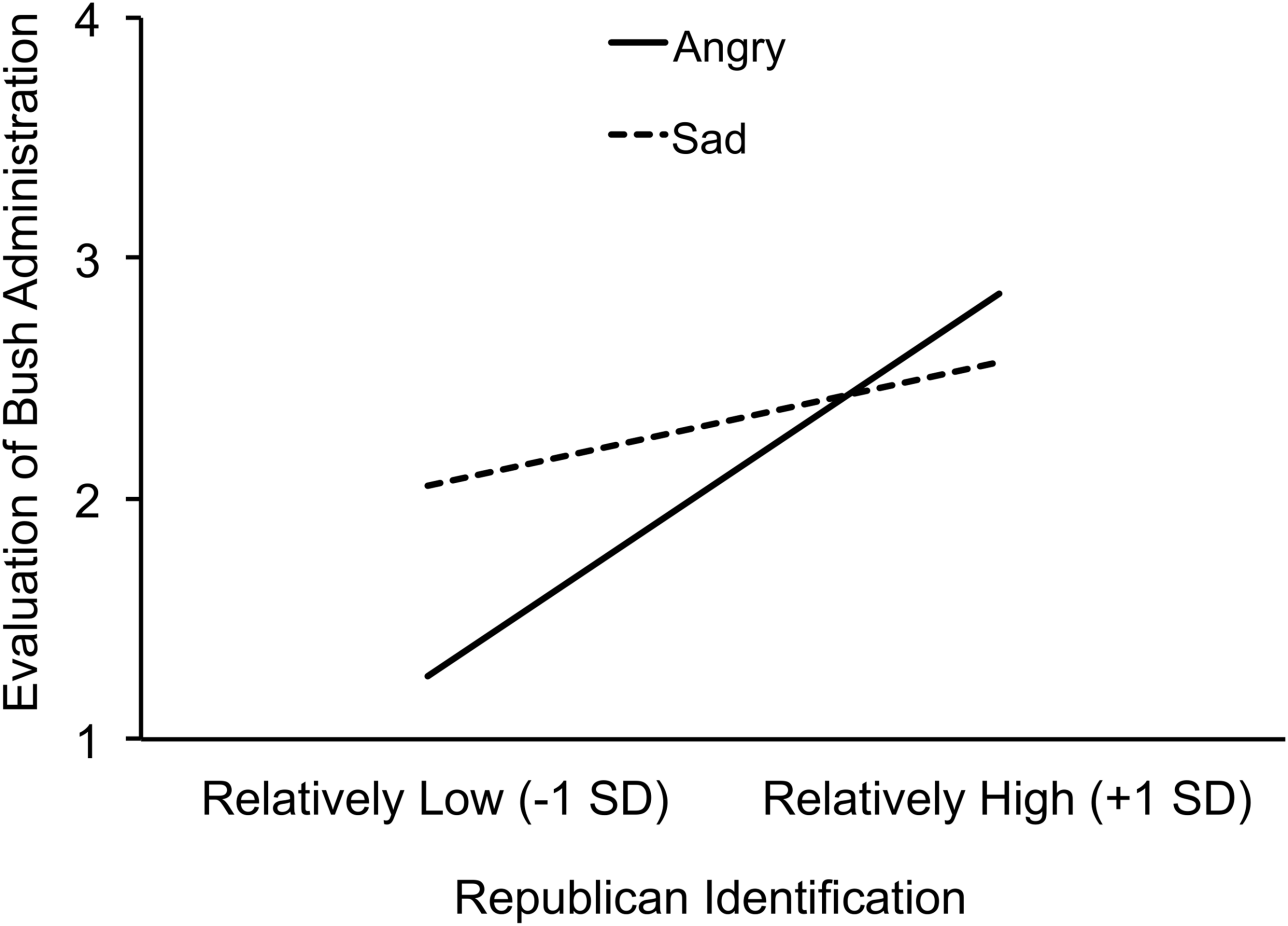
Study 1: Emotion condition and issue partisanship. The association between Republican identification (x-axis) and evaluation of the Bush Administration’s response to Hurricane Katrina (y-axis) is stronger in the anger condition (solid line) than in the sadness condition (dashed line). Note that slopes are plotted at –1 and +1 SD on Republican Party identification. This corresponds to 0.85 and 4.83 on the scale ranging from 1 = *not very strong* to 7 = *very strong* to measure Republican Party identification.

Consistent with hypothesis 1 (issue partisanship), anger increased the association between how strongly people identified with the Republican Party and positive evaluation of the Bush Administration’s response to Hurricane Katrina. There was a significant interaction between Republican identification and emotion condition, *b* = .12, *t*(108) = 2.21, *p* = .029, partial η^2^ = .04 (see Fig 2). In the anger condition, the association between Republican identification and evaluation of the Bush Administration was stronger, *b* = .42, *t*(108) = 4.82, *p* < .001, partial η^2^ = .18, than it was in the sadness condition, *b* = .17, *t*(108) = 2.16, *p* = .033, partial η^2^ = .04. [In both sets of regression analyses (for testing hypothesis 1 and hypothesis 2) in this study we explored the interaction between Democratic identification and emotion condition. The interaction was not significant in either case, *t*’s < 1.]

**Fig 3:**
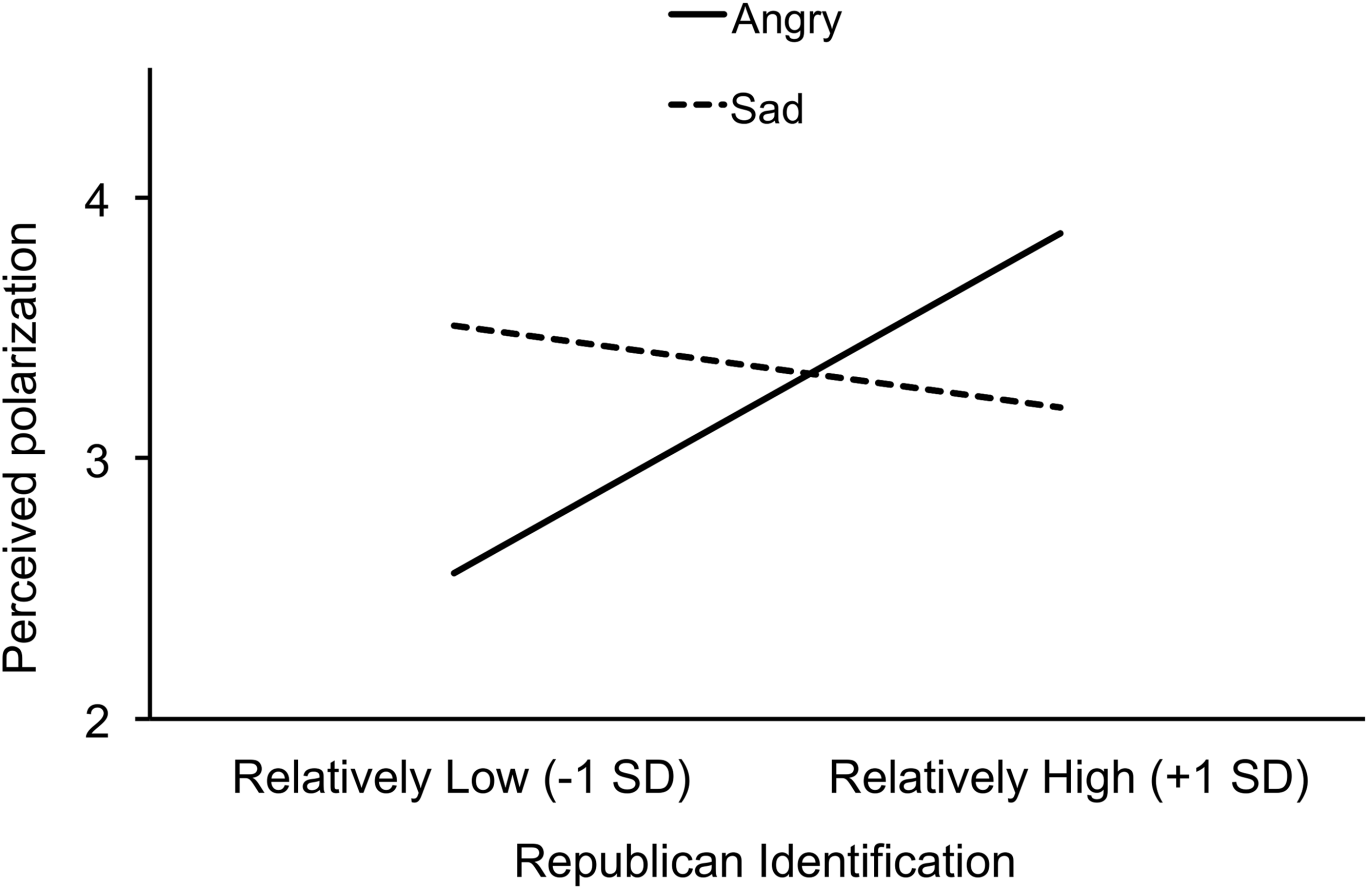
Study 1: Emotion condition and partisan perceived polarization. The association between Republican identification (x-axis) and perceived polarization between Democrats’ and Republicans’ evaluation of the Bush Administration’s response to Hurricane Katrina (y-axis) is stronger in the anger condition (solid line) than in the sadness condition (dashed line).

To test hypothesis 2, we regressed perceived polarization (operationalized as above) on Republican identification (mean centered), emotion condition (anger = +1, sadness = –1), the Republican identification × emotion condition interaction, Democratic identification, and participants’ own evaluation of the Bush Administration (see Table 1). As predicted, the interaction was significant, *b* = .20, *t*(108) = 2.07, *p* = .040, partial η^2^ = .04 (see Fig 3). [We also tested the interactions for both hypotheses without controlling for Democratic identification. When predicting evaluation of the Bush Administration, the coefficient for the interaction between Republican identification and emotion condition is *b* = .10, *t*(109) = 1.65, *p* = .102, partial η^2^ = .04. When predicting perceived polarization, the coefficient for the interaction between Republican identification and emotion condition is *b* = .22, *t*(109) = 2.31, *p* = .023, partial η^2^ = .05.] The association between Republican identification and perceived polarization was stronger in the anger condition, *b* = .33, *t*(108) = 1.99, *p* = .049, partial η^2^ = .03, than in the sadness condition, *b* = –.08, *t* < 1, *ns*.

These results indicate that, compared with incidental sadness, anger increased the strength of associations between Republican identification and evaluation of the Bush Administration and perceived polarization between Democrats and Republicans.

These results are consistent with the hypotheses that to the degree people identify with a political group that is likely to experience threat—both because Republicans were publically criticized regarding Hurricane Katrina and because identification as a Republican is associated with sensitivity to threat—incidental anger increases issue partisanship (hypothesis 1) and partisan perceived polarization (hypothesis 2). One limitation of this study is the lack of a manipulation check for the emotion induction. In Study 2, we sought to replicate and expand these findings in three ways. First, because Study 1 did not include a neutral emotion condition, an important unanswered question is whether anger increased these associations with Republican identification or sadness decreased these associations. Second, because we had no manipulation check in Study 1, we included an emotion manipulation check in Study 2. Third, we sought to replicate these findings in the context of a different national tragedy to increase the generalizability of our findings.

## Study 2: Tucson, AZ, Mass Shooting

We conducted the study shortly after Jared Lee Loughner shot 19 people in an attempted assassination of U.S. Representative Gabrielle Giffords (Democrat) on 8 January 2011 in Tucson, Arizona. Political rancor following the shooting emphasized the role that “hateful” political speech—in particular, Republican Sarah Palin’s political campaign showing a map with crosshairs targeting several congressional seats including that of Representative Giffords—played in fostering a climate of violence. We reasoned that this context, where a prominent Republican faced intense public criticism and blame, threatened people to the degree that they identify with the Republican Party, which should lead to stronger intergroup conflict related reactions.

Accordingly, we expected that, compared with incidental sadness and the neutral control condition, anger would increase the associations of strength of identification with the Republican Party with (1) less agreement that Palin’s actions contributed to the shooting and with (2) perceived polarization between Democrats and Republicans. We included a neutral control condition to allow us to distinguish whether anger increased these associations, as we predicted, or sadness decreased these associations.

### Method

We conducted the study between 20 and 25 January 2011, approximately two weeks after the 8 January 2011 mass shooting. Participants from Mechanical Turk (*N* = 303, 61% female) participated in exchange for $1. Ethical approval was obtained from the institutional review board at the University of Colorado Boulder. The institutional review board waived the need for written informed consent from the participants. Participants varied in age (*M* = 34.28 years, *SD* = 11.39, *IQR* = 25–43 years) and ethnicity (White = 84%, Hispanic = 2%, Asian American = 6%, African American = 5%, other = 3%).

We randomly assigned participants to the anger (*n* = 91), sadness (*n* = 89), or neutral conditions (*n* = 119). The emotion induction was identical to Study 1. In the neutral condition, participants were asked to describe “activities you performed yesterday.” We excluded four participants from analyses because they did not follow the instructions; instead of describing what made them angry or sad, they described neutral activities (resulting *n* = 299).

As an emotion manipulation check, participants reported how much (1 = *very slightly or not at all*, 2 = *a little*, 3 = *moderately*, 4 = *quite a bit*, 5 = *extremely*) they felt several emotions “at the present moment.” Three emotions (angry, mad, enraged) centered on anger (*α* = .96), and six (sad, distressed, blue, downhearted, alone, lonely) on sadness (*α* = .93).

As a measure of participants’ personal blame of Sarah Palin, they indicated their agreement (1 = *strongly disagree*, 7 = *strongly agree*) with the statement, “Sarah Palin contributed to a climate of political violence, which may have led to violent acts such as the Arizona shooting.” To measure perceived polarization, participants estimated, separately and in counterbalanced order, how much the typical Republican and how much the typical Democrat agreed with the same statement. We subtracted estimates of the typical Republican’s blame of Palin (*M* = 2.11, *SD* = 1.38) from estimates of the typical Democrat’s blame of Palin (*M* = 5.49, *SD* = 1.43) such that higher numbers reflect greater perceived polarization.

**Fig 4:**
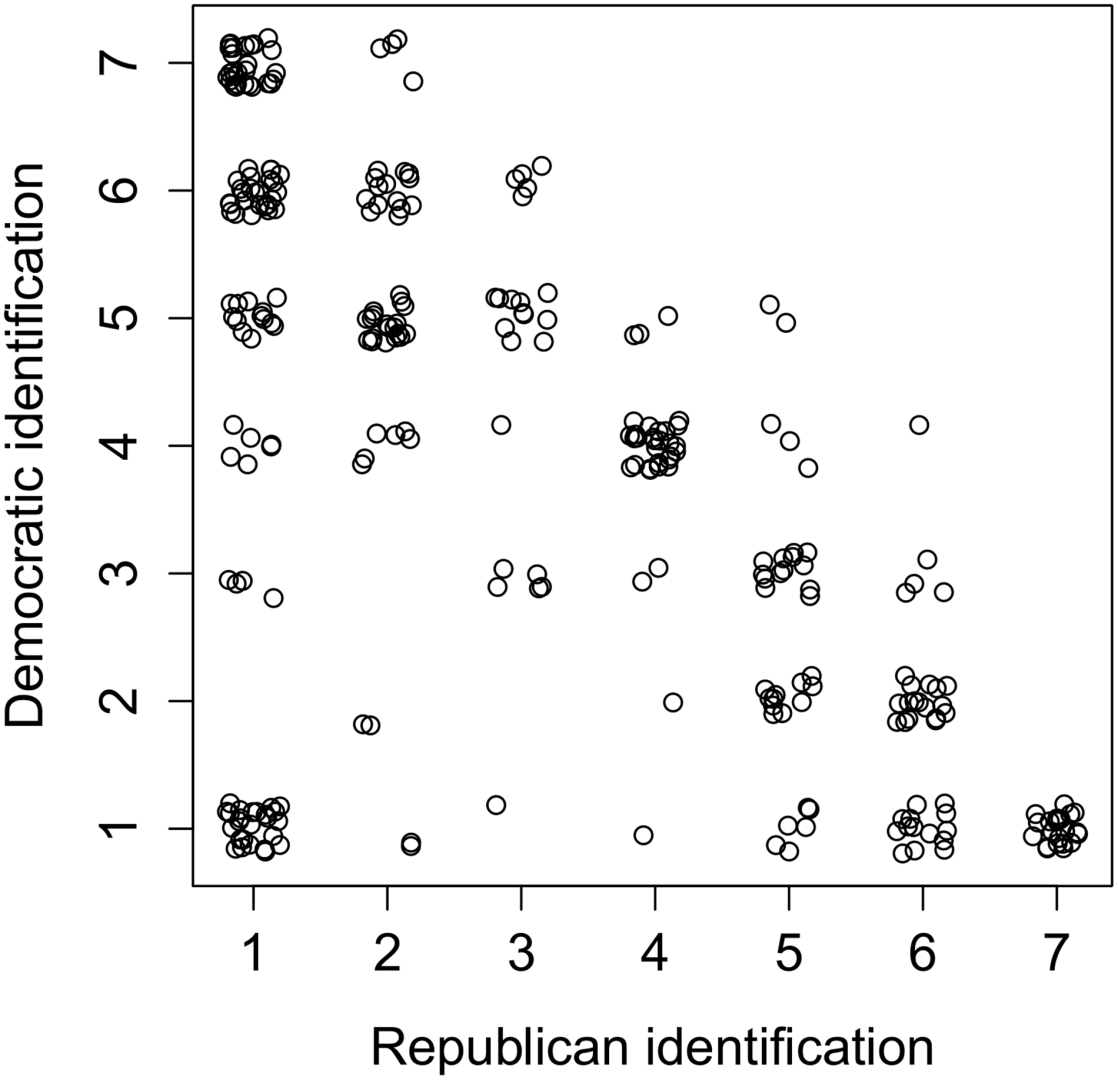
Study 2: Scatterplot displaying Republican and Democratic identification. The scatterplot displays variance on Republican identification (x-axis) and Democratic identification (y-axis) in the sample for Study 2 (*N* = 299).

We measured political identification as in Study 1. The two measures were negatively correlated (*r* = –.61, see Fig 4). Participants identified more strongly as Democrat (*M* = 3.77, *SD* = 2.08) than Republican (*M* = 3.16, *SD* = 2.09), *t*(298) = 2.80, *p* = .005, *d* = .16. Similar to Study 1, the sample for Study 2 had respondents across the full spectrum of Republican identification (see Fig 4). After completing all measures, participants were offered a written debriefing.

### Results and Discussion

The authors confirm that all data underlying the findings are fully available without restriction. We confirm that we reported all conditions and data exclusions. We collected additional measures that were not directly related to the hypotheses we tested here. All measures are included in the supplementary material. The sample size for this study was based on how much data we could afford to collect. Data may be accessed from Figshare under the DOI: http://dx.doi.org/10.6084/m9.figshare.1464979.

To examine whether the emotion induction affected participants’ reported emotions, we conducted a 3(emotion condition: anger, sadness, neutral) × 2(reported emotion: anger, sadness) ANOVA with repeated measures on the second factor. A main effect of emotion condition, *F*(2, 296) = 26.65, *p* < .001, partial η^2^ = .15, reflected that participants in the anger (*M* = 2.14, *SD* = 1.03) and sadness (*M* = 1.94, *SD* = 0.95) conditions reported more intense emotions than did participants in the neutral condition (*M* = 1.34, *SD* = 0.52), *F*(1, 297) = 50.66, *p* < .001, partial η^2^ = .15. There was also a significant interaction, *F*(2, 296) = 8.49, *p* < .001, partial η^2^ = .05 (see Table 2). Participants in the anger condition reported higher levels of anger (*M* = 2.17, *SD* = 1.22) compared with the neutral condition (*M* = 1.15, *SD* = 0.50), *F*(1, 208) = 68.53, *p* < .001, partial η^2^ = .25, and compared with the sadness condition (*M* = 1.73, *SD* = 1.13), *F*(1, 178) = 6.42, *p* = .012, partial η^2^ = .03. Participants in the sadness condition reported higher levels of sadness (*M* = 2.16, *SD* = 0.97) compared with the neutral condition (*M* = 1.53, *SD* = 0.68), *F*(1, 206) = 30.81, *p* < .001, partial η^2^ = .13, but not compared with the anger condition (*M* = 2.10, *SD* = 1.17), *F* < 1, *ns*. The emotion manipulation thus changed the overall profile of participants’ self-reported emotional arousal.

**Table 2:** Study 1: Emotion manipulation check.

		*Reported Emotion*	
		Anger	Sadness
*Emotion Condition*	*Anger (n = 91)*	2.17_a_ (1.22)	2.10_a_ (1.17)
	*Sadness (n = 89)*	1.73_b_ (1.13)	2.16_a_ (0.97)
	*Neutral (n = 119)*	1.15_c_ (0.50)	1.53_b_ (0.68)

*Average self-reported emotion (standard deviation in parentheses) after describing autobiographical events that made participants feel angry*, *sad*, *or neutral*.

^a^ Means with different subscripts within each column are significantly different, *p* < .05.

^b^ Means with different subscripts within each column are significantly different, *p* < .05.

^c^ Means with different subscripts within each column are significantly different, *p* < .05.

To examine the effect of anger compared with sadness and neutrality on issue partisanship and partisan perceived polarization, we created two orthogonal contrasts. The Anger contrast compared anger (+2) with the sadness (–1) and neutral (–1) conditions; the Sadness contrast compared the sadness (+1) and neutral (–1) conditions (anger = 0).

We regressed participants’ blame of Palin on Republican identification (mean centered), the Anger contrast, the Sadness contrast, the interactions between Republican identification and each of the Anger and Sadness contrasts, Democratic identification, and perceived polarization (operationalized as described above, see Table 3). [In Study 2, own attitudes and perceived polarization were not correlated, *r* = .02. Thus, we conducted the same two regression analyses without controlling for perceived polarization when testing hypothesis 1 and without controlling for own attitudes when testing for hypothesis 2. These analyses revealed the same pattern of significant results as the regression models reported here. To keep the regression models for Study 1 and Study 2 as similar as possible, we will only provide detailed reports for the regression models controlling for perceived polarization (hypothesis 1) and controlling for own attitudes (hypothesis 2).] Consistent with Study 1, Republican identification was negatively associated with blame of Sarah Palin, *b* = –.38, *t*(291) = –6.20, *p* < .001, partial η^2^ = .12, and independently, Democratic identification was positively associated with blaming Sarah Palin, *b* = .25, *t*(291) = 4.12, *p* < .001, partial η^2^ = .05.

**Table 3:** Study 2: Linear regression models.

*Dependent Variables and* Predictors	*b*	*SE(b)*	*t*	*p*	95% CI	partial η^2^
*Personal Blame of Palin*						
Anger contrast	.02	.07	0.33	.744	–.12, .17	.01
Sadness contrast	–.01	.12	–0.04	.967	–.25, .24	.01
Republican identification (centered)	–.38	.06	–6.20	< .001	–.50,–.26	.12
Anger × Republican identification	–.06	.04	–1.78	.076	–.13, .01	.01
Sadness × Republican identification	–.02	.06	–0.36	.717	–.14, .10	.01
Democratic identification	.25	.06	4.12	< .001	.13, .38	.05
Perceived polarization	.05	.05	1.09	.278	–.04, .15	.01
*Perceived Polarization*						
Anger contrast	.07	.09	0.71	.476	–.11, .24	.01
Sadness contrast	–.21	.15	–1.40	.161	–.51, .08	.01
Republican identification (centered)	.04	.08	0.52	.602	–.12, .20	.01
Anger × Republican identification	.10	.04	2.31	.022	.01, .18	.02
Sadness × Republican identification	–.03	.07	–0.41	.685	–.17, .11	.01
Democratic identification	–.05	.08	–0.70	.486	–.21, .10	.01
Personal Blame of Palin	.08	.07	1.09	.278	–.06, .22	.01

*Linear regression models estimating personal blame of Sarah Palin for fostering a climate of violence (top panel) and perceptions of polarization between Democrats and Republicans (bottom panel)*. Republican identification is mean centered. Anger contrast: anger = +2, sadness = –1, neutral = –1. Sadness contrast: anger = 0, sadness = +1, neutral = –1.

**Fig 5:**
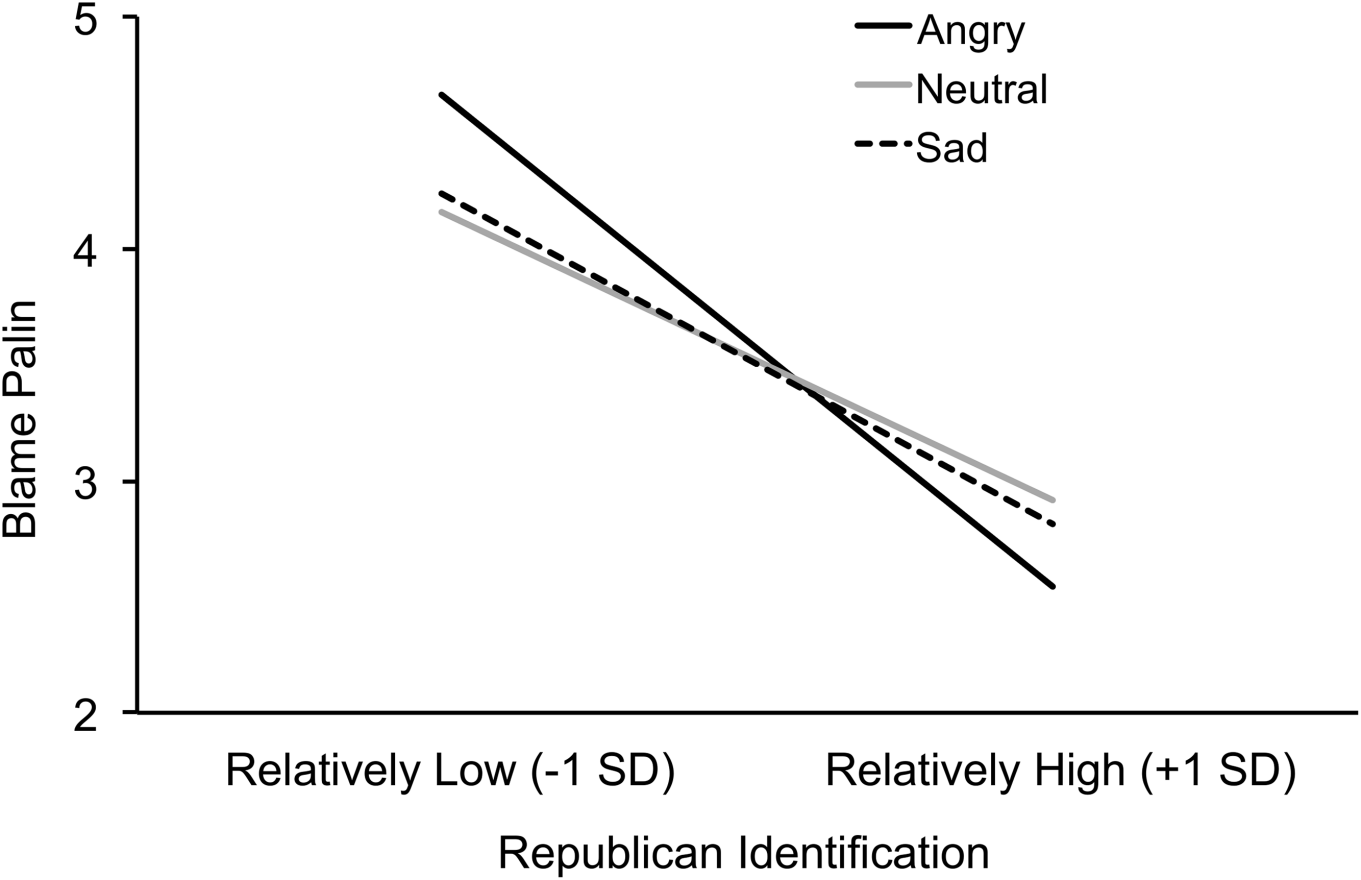
Study 2: Emotion condition and issue partisanship. Republican identification (x-axis) is negatively associated with partisan blaming of Sarah Palin for fostering a climate of violence that contributed to the Tucson, AZ, shooting (y-axis), and this association tends to be stronger in the anger condition (solid black line) than in the neutral condition (light grey line) and sadness condition (dashed line). Note that slopes are plotted at –1 and +1 SD on Republican Party identification. This corresponds to 1.07 and 5.25 on the scale ranging from 1 = *not very strong* to 7 = *very strong* to measure Republican Party identification.

Consistent with hypothesis 1, the predicted interaction between Republican identification and the Anger contrast was marginally significant, *b* = –.06, *t*(291) = –1.78, *p* = .076, partial η^2^ = .01 (see Fig 5). The association between Republican identification and blaming Sarah Palin was (descriptively) more negative in the anger condition, *b* = –.51, *t*(293) = –5.39, *p* < .001, partial η^2^ = .09, than in the sadness condition, *b* = –.34, *t*(293) = –3.55, *p* < .001, partial η^2^ = .04, and than in the neutral condition, *b* = –.30, *t*(293) = –3.39, *p* < .001, partial η^2^ = .04. The interaction between Republican identification and the Sadness contrast was not significant, *t* < 1. The pattern of results thus conceptually replicated Study 1, suggesting that anger increases the association between Republican identification and endorsement of group-protective attitudes, rather than sadness decreasing this association.

**Fig 6:**
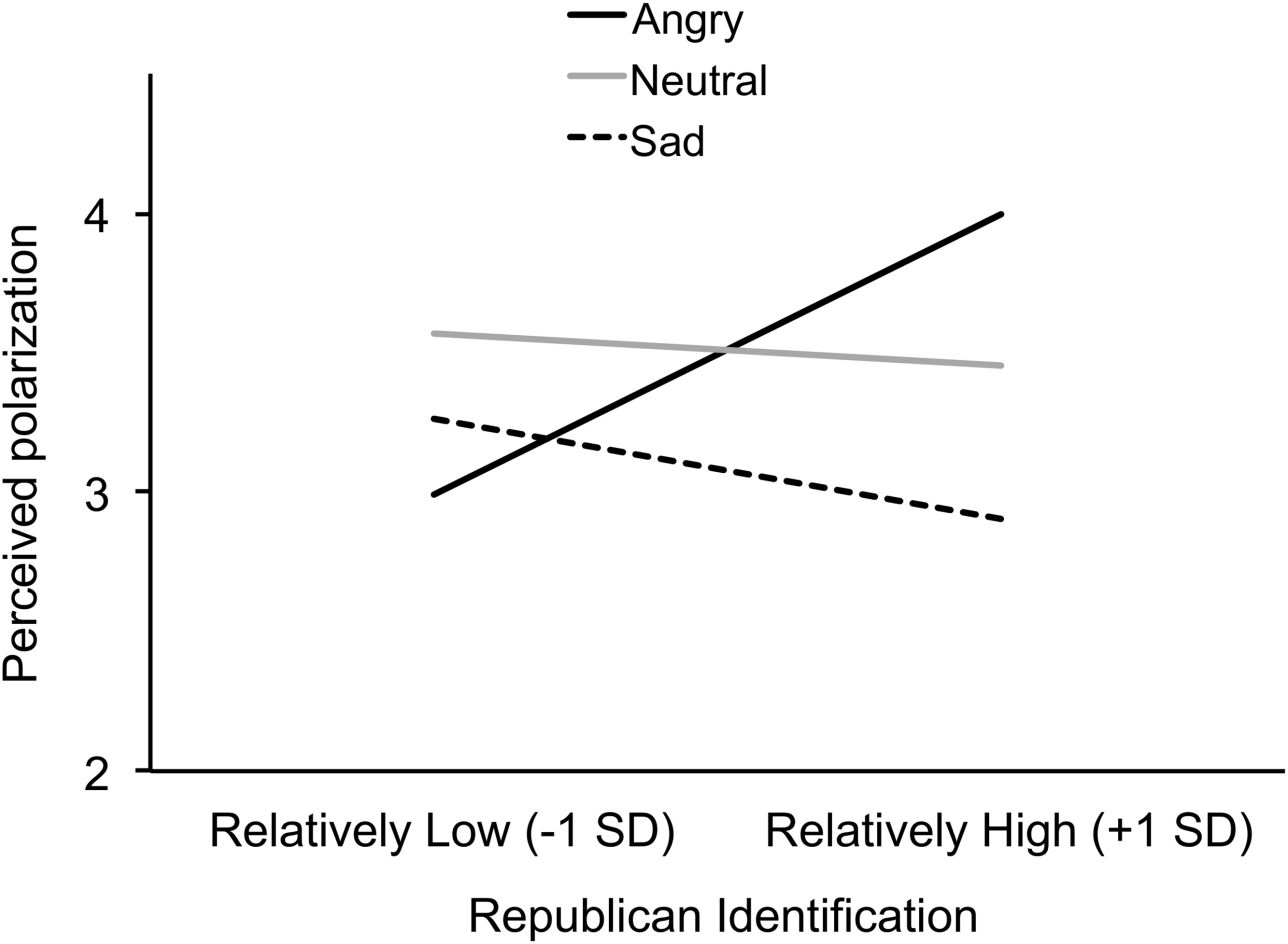
Study 2: Emotion condition and partisan perceived polarization. Republican identification (x-axis) is positively associated with perceived polarization in blaming of Sarah Palin (y-axis), and this association is stronger in the anger condition (solid black line) than in the neutral condition (light grey line) and sadness condition (dashed line).

To test hypothesis 2, we regressed participants’ perceptions of polarization (operationalized as above) on Republican identification (mean centered), the Anger contrast (defined above), the Sadness contrast (defined above), the interactions between Republican identification and each of the Anger and Sadness contrasts, Democratic identification, and personal blaming of Palin (see Table 3). As predicted, there was a significant interaction between Republican identification and the Anger contrast, *b* = .10, *t*(291) = 2.31, *p* = .022, partial η^2^ = .02. [We also tested the interactions for both hypotheses without controlling for Democratic identification. When predicting blame of Sarah Palin, the coefficient for the interaction between Republican identification and Anger contrast is *b* = –.06, *t*(292) = –1.73, *p* = .085, partial η^2^ = .01. When predicting perceived polarization, the coefficient for the interaction between Republican identification and Anger contrast is *b* = .10, *t*(292) = 2.29, *p* = .022, partial η^2^ = .02.] In the anger condition, Republican identification was more positively associated with perceived polarization, *b* = .24, *t*(293) = 2.00, *p* = .047, partial η^2^ = .01, than in the sadness, *b* = –.09, *t* < 1, and neutral conditions, *b* = –.03, *t* < 1 (see Fig 6). [In both sets of regression analyses, we also explored the interactions between Democratic identification and each of the Anger and Sadness contrasts. The interactions were not significant in either case, *t*(289) = –1.42, *ns*, for political polarization, and *t*(289) = –1.17, *ns*, for perceived political polarization.] Conceptually replicating and extending Study 1, anger increased the association between Republican identification and perceived polarization, but sadness did not reduce this association.

These results demonstrate that incidental anger increased the association of strength of Republican identification with disagreement that Palin contributed to a violent climate and with perceived polarization between Democrats and Republicans. The inclusion of a neutral control condition indicated that anger exacerbated these relationships, but sadness did not reduce the relationships. This finding adds additional evidence that incidental anger exacerbates group protective social cognition.

## General Discussion

The results of two studies with diverse samples in the United States demonstrated that incidental anger increases the associations of Republican identification with endorsement of partisan attitudes and with perceived polarization between Democrats and Republicans. We observed these patterns in the contexts of two national tragedies, the aftermath of Hurricane Katrina in 2005 and the mass shootings in Tucson, Ariz., in 2011. Both contexts were points of public debate and flashpoints of political intergroup conflict.

To our knowledge, these are the first studies to demonstrate that incidental anger increases both issue partisanship and partisan perceived polarization. Our findings use measures of reactions to intergroup threat—the effect of political party identification on attitude polarization and on perceived attitude polarization—in the context of naturally occurring groups and genuine political conflicts beyond the confines of the laboratory. These findings thus provide important evidence that background emotions can exacerbate partisan divisions and perceptions of political polarization in everyday life.

One question is whether the effect of anger on perceived polarization, which we operationalized as the difference between Democrats and Republicans, is primarily attributable to perceptions of Democrats, perceptions of Republicans, or both. It may be, for example, that anger increases the negative association between identification as a Republican and perception of Democrats as an opposing group. To examine this question, we repeated our analyses of perceived polarization with perceptions of Democrats and perceptions of Republicans as separate dependent variables. The key interactions were in the predicted direction and of comparable magnitude both for perceptions of Democrats (Study 1: *b* = –.11, *t*(108) = –1.91, *p* = .058; Study 2: *b* = .05, *t*(291) = 1.79, *p* = .074) and for perceptions of Republicans (Study 1: *b* = .09, *t*(108) = 1.27, *p* = .207; Study 2: *b* = –.05, *t*(291) = –1.81, *p* = .071). That these interaction coefficients were not statistically significant is not surprising given that the dependent variable included only one half of the perceived polarization difference score. The effect of anger on perceived polarization is thus attributable to perceptions of Democrats and of Republicans.

Of course, by relying on naturally occurring groups and national tragedies, some sacrifice is made in control over the definition of groups and events. We predicted that incidental anger would increase the associations of identification with the Republican Party (but not Democratic) with partisan attitudes and perceived polarization. Both contexts that we used—the aftermath of Hurricane Katrina and criticism of Sarah Palin for fostering violence—entailed strong critiques of salient Republican leaders. We hypothesized that anger would interact with Republican identification both because conservatives (who tend to identify as Republican) are more sensitive to threat [30,31] and because Republicans were targets of public criticism in both national tragedies.

One assumption that we were not able to test in the context of our studies is whether incidental anger would exert an impact on people’s judgments in contexts where Democratic leaders are sharply criticized. One prediction would be that situational contexts where Democratic leaders face intense public criticism would pose a threat to the degree people identify with the Democratic Party. This would lead to an interaction between emotion and identification with the Democratic Party. However, another prediction would be that—even in contexts where Democrats are under attack—people might show more issue partisanship and perceived polarization to the degree they identify with the Republican Party because of generally higher levels of threat sensitivity [30,31]. Thus, in such contexts, we would predict that anger interacts with both identifications.

Future research might also more carefully tease apart the different components of incidental emotion manipulation. The results of the emotion manipulation check in Study 2 suggest that although participants reported more anger in the incidental anger condition than in the sadness or neutral condition, they did not report significantly more anger than sadness, and they did not report less sadness than did participants in the incidental sadness condition (see Table 2). On the one hand, this pattern implies that our incidental emotion manipulation was relatively imprecise. On the other hand, this pattern implies that our study was a conservative test of our hypotheses. To the extent that our manipulation confounded anger and sadness, that would have reduced the effect of incidental anger compared with incidental sadness. Yet as can be seen in Figs 5 and 6, the results in the incidental anger condition were different from both the incidental sadness and neutral conditions. Further, the results of the incidental sadness condition were not different from the neutral condition. If anything, the confounding of anger and sadness would dampen rather than artificially inflate differences between conditions.

Future research could also clarify the processes by which incidental anger exerts its effects. Based on previous research (e.g., [5,10]), we believe that because anger is associated with intergroup conflict, arousal of incidental anger increases identity related reactions to intergroup threat. An alternative possibility is that incidental anger increases the perceived magnitude of threat, or that it increases shallow, heuristic processing [7,9,45].

Despite these lingering ambiguities, we believe that the effects of anger in the present studies are important for two reasons. First, anger might contribute to a vicious cycle in which anger increases partisan attitudes and perceptions of polarization, which then further increases anger and perceptions of group conflict [46]. This is especially noteworthy and worrying considering that anger can increase support for aggressive actions and policies [47]. Second, political elites and the media often include anger eliciting content in their communications—at least during specific political events or when discussing specific political issues such as terrorism [48,49]. Although these strategies might “energize the base,” our research suggests such anger inductions might also increase both actual and perceived partisan differences.

In conclusion, anger is sometimes lauded as a motivating force for civic action. Citizens and politicians whose righteous anger fuels action are held as admirable examples of a functional democracy. We think the present results imply that such admiration of anger can be misplaced. Being angry can increase the political divide—and perceptions of the political divide—which may pose a barrier to intergroup cooperation, compromise, and democratic functioning.
